# PI3K/mTOR inhibitor omipalisib prolongs cardiac repolarization along with a mild proarrhythmic outcome in the AV block dog model

**DOI:** 10.3389/fcvm.2022.956538

**Published:** 2022-08-03

**Authors:** J. J. A. van Bavel, C. Pham, H. D. M. Beekman, M. J. C. Houtman, A. Bossu, R. W. Sparidans, M. A. G. van der Heyden, M. A. Vos

**Affiliations:** ^1^Division of Heart and Lungs, Department of Medical Physiology, University Medical Center Utrecht, Utrecht, Netherlands; ^2^Division Pharmacology, Department of Pharmaceutical Sciences, Faculty of Science, Utrecht University, Utrecht, Netherlands

**Keywords:** omipalisib, AV block dog model, QT prolongation, PI3K inhibition, ventricular arrhythmia (VA)

## Abstract

**Background:**

The phosphoinositide 3-kinase (PI3K) signaling pathway is an interesting target in cancer treatment. The awareness of the proarrhythmic risk of PI3K inhibitors was raised because PI3K is also involved in regulating signaling toward cardiac ion channels. Canine cardiomyocytes treated with PI3K inhibitors show an increased action potential duration and reduced cardiac repolarizing currents. Now, the potential proarrhythmic effect of chronic treatment of PI3K/mTOR inhibitor GSK2126458 (omipalisib) was investigated in the atrioventricular (AV) block dog model.

**Methods:**

Purpose-bred Mongrel dogs received complete AV block by ablation of the bundle of His and their hearts were paced in the right ventricular apex at VDD-mode (RVA-VDD). In this way, sinus rhythm was maintained for 15 ± 1 days and thereby bradycardia-induced cardiac remodeling was prevented. Dogs received 1 mg/kg omipalisib once (*n* = 3) or twice (*n* = 10) a day *via* oral administration for 7 days. Under standardized conditions (anesthesia, bradycardia at 60 beats/min, and a dofetilide challenge), potential proarrhythmic effects of omipalisib were investigated.

**Results:**

Twice daily dosing of omipalisib increased accumulative plasma levels compared to once daily dosing accompanied with adverse events. Omipalisib prolonged the QT interval at baseline and more strongly after the dofetilide challenge (490 ± 37 to 607 ± 48 ms). The arrhythmic outcome after omipalisib resulted in single ectopic beats in 30% of dogs perpetuating in multiple ectopic beats and TdP arrhythmia in 20% of dogs. Isolated ventricular cardiomyocytes from omipalisib-treated dogs showed a diminished I_Ks_ current density.

**Conclusion:**

Chronic treatment of PI3K/mTOR inhibitor omipalisib prolonged the QT interval in a preclinical model under standardized proarrhythmic conditions. Furthermore, this study showed that electrical remodeling induced by omipalisib had a mild proarrhythmic outcome.

## Introduction

The PI3K signaling pathway is involved in a wide range of cellular processes important for cell growth, cell proliferation, cell survival and autophagy (1). Enhanced activity of the PI3K signaling pathway is a hallmark of a broad spectrum of human cancers, associated with increased angiogenesis and cell survival. Mutations in genes of members of the PI3K pathway, such as *PIK3CA* (p110α), *AKT*, and *PTEN* have been found in various cancer types (2, 3). Aside from cancer, PI3K signaling is involved in triggering other human diseases, such as tuberous sclerosis and psoriasis (4–6). Certainly, targeting PI3K pathway elements is of interest in the development of therapeutic interventions (7). Chemotherapeutic agents that target or interfere with PI3K signaling include pan-PI3K inhibitors (targeting p110 isoforms), isoform-specific PI3K inhibitors, and dual PI3K/mTOR inhibitors, which are tested in clinical trials alone or in combined conditions (8). Though, the significant role of the complex PI3K signaling pathway in functioning of the cell is accompanied with challenges in the development of therapeutic interventions that aim to target the pathway. Combination therapies are shown to be more effective than monotherapy but bring complexity issues and adverse effects, such as drug-related toxicity and compensatory signaling, resulting in resistance causing failure of the drug in clinical trials (9).

Cardiovascular toxicity is a side effect of anti-cancer treatment that emerged the cardio-oncology discipline (10). Regulation of the PI3K signaling pathway in the cardiovascular research field concerns crucial cellular processes such as survival and autophagy (11, 12). Adverse cardiac effects accompanied with PI3K inhibition include a reduction in adaptive responses (e.g., hypertrophy and angiogenesis) to pathologic stressors, and a higher risk for cardiovascular disease in diabetic patients due to reduced glucose oxidation (13). In terms of cardiac electrophysiology, the PI3K pathway is involved in regulating signaling toward cardiac ion channels (14). Transgenic mice with cardiac-specific constitutively active PI3Kα showed upregulated mRNA levels of ion channels responsible for the slow delayed rectifier current (*I*_Ks_), sodium current (*I*_Na_) and calcium current (*I*_Ca, L_) (15). Suppression of PI3K signaling in isolated dog cardiomyocytes for 2 h prolonged the action potential duration (APD), reduced rapid delayed rectifier current (*I*_Kr_), *I*_Ks_, *I*_Ca,L_ and *I*_Na−peak_ densities, and enhanced *I*_Na−late_ density (16). Furthermore, the decreased PI3K signaling caused prolongation of the QT interval recorded from isolated hearts of PI3Kα^−/−^ mice and isolated hearts of wildtype mice perfused with tyrosine kinase inhibitor nilotinib (16). We aimed to investigate the potential proarrhythmic effect of chronic inhibition of the PI3K pathway in a preclinical animal model.

GSK2126458 (omipalisib) was presented in 2010 as dual PI3K/mTOR inhibitor (17). It is highly active against all PI3K isoforms and both mTOR complexes and it is more potent than BEZ235 and GDC-0941 (17). Its promising role as potential therapeutic agent is shown in *in vitro* studies reflecting a variety of cancer types and idiopathic pulmonary fibrosis (18–21). Chronic treatment of Tsc2^+/−^ mice, a model of tuberous sclerosis, with omipalisib reduced the number and size of solid renal tumors (22). The compound is currently tested in phase I clinical trials, and to date, three publications have documented drug tolerance, dosing safety, clinical outcome, and drug combination effectivity in human patients (23–25). ECG parameters were addressed in one article, and besides a clinically insignificant reduced heart rate, no cardiac abnormalities were reported as adverse events after omipalisib treatment (25).

The sensitive atrioventricular (AV) block dog model has been used for the last three decades to test potential pro- and antiarrhythmic effects of pharmacological compounds (26). A combination of AV block-induced cardiac remodeling, anesthesia, bradycardia, and infusion of *I*_Kr_ blocker dofetilide as the final hit, predisposes the heart to Torsade de Pointes (TdP) ventricular arrhythmias (27). The electrical component of cardiac remodeling includes a reduced repolarization reserve, which is caused by a downregulation of *I*_Kr_ and *I*_Ks_ and results in QT prolongation (28, 29). It is yet unclear whether diminished ion current densities induced by chronic PI3K inhibition result in proarrhythmic conditions. Therefore, we investigated the proarrhythmic risk of PI3K/mTOR inhibitor omipalisib by replacing AV block-induced electrical remodeling by chronic omipalisib treatment.

## Materials and methods

### Animals

Animal handling and care were in accordance with the Directive 2010/63/EU of the European Parliament and of the Council of 22 September 2010 on the protection of animals used for scientific purposes and the Dutch law on animal experimentation. Experiments were approved by the Central Authority for Scientific Procedures on Animals. Dogs were housed in pairs in kennels containing wooden bedding material, had ad libitum access to drinking water and received food pellets twice a day. The animals were allowed to play outside once a day and their welfare was checked daily.

### Animal preparation

Adult purpose-bred mongrel dogs (*n* = 13, Marshall, New York, USA) were included in the experimental protocol. The dogs were fasted overnight and received premedication (0.02 mg/kg atropine, 0.5 mg/kg methadone, and 0.5 mg/kg acepromazine i.m.) 30 min prior to the surgical procedure. General anesthesia was induced by sodium pentobarbital (Nembutal, 25 mg/kg i.v.) and maintained by 1.5% isoflurane in O_2_ and N_2_O (1:2 ratio) *via* mechanical ventilation at 12 breaths/min. To minimize pain and the risk of inflammation, dogs received analgesics (0.1 mg/kg Metacam s.c. before surgery and 0.3 mg Temgesic i.m. after surgery) and antibiotics (1,000 mg ampicillin i.v. before and i.m. after surgery).

### Control experiment

**Figure 1:**
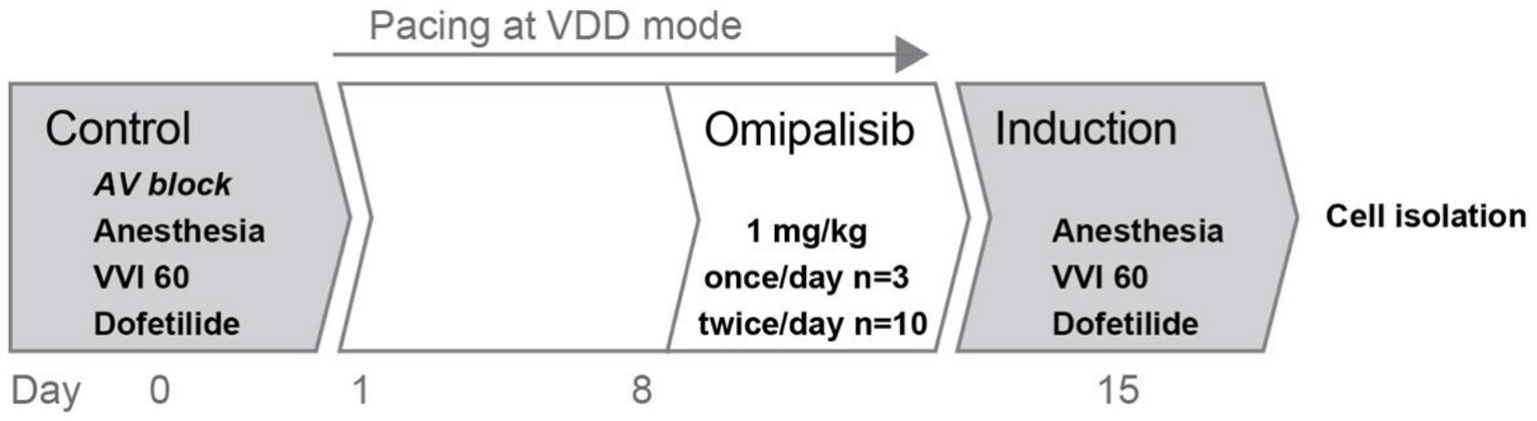
Overview of the experimental protocol. Two experiments were performed in series: control and induction, before and after omipalisib treatment to examine the potential proarrhythmic effect of omipalisib in the AV block dog model.

Two serial experiments were performed under general anesthesia with each dog serving as its own control (Figure 1). The first experiment under general anesthesia included implantation of a pacemaker device (Medtronic, Maastricht, The Netherlands) with a right atrial lead sensing native atrial activity and a screw-in lead for stimulation of the right ventricular (RV) apex. A transmural needle biopsy was obtained from the left ventricular (LV) apex and immediately frozen using liquid nitrogen for later protein analysis. Radiofrequency ablation of the His bundle induced complete and irreversible AV block to induce bradycardia. The arrhythmic status of the heart at this control time point was tested according to our standardized protocol: under anesthesia, at bradycardia (continuous RV pacing at 60 bpm, VVI 60), and infusion of I_Kr_ blocker dofetilide (Biorbyt, dissolved in 100 μl 0.1 M HCl and diluted in saline, 25 μg/kg i.v.) for 5 min or until the first TdP arrhythmia occurred.

### Pacing at VDD mode

After the control experiment, AV block-induced cardiac remodeling on a structural, electrical, and contractile level was prevented by continuous pacing at VDD mode (electrical conduction sensed in the atria was perpetuated *via* pacing in the RV apex). In this way, sinus rhythm was resembled and maintained until the induction experiment.

### Chronic treatment with omipalisib

Eight days after the control experiment, dogs received 1 mg/kg omipalisib daily for 7 days (Figure 1). First, three dogs (all females, bodyweight: 21 ± 2 kg, age: 12 ± 0 months at control experiment) received omipalisib once a day at 8:00 AM, based on results from a human clinical trial (23). For optimization of target inhibition across a 24-h interval and omipalisib concentration in plasma >30 ng/ml (17, 23, 25), dosing frequency was increased to twice a day for the next group. Ten dogs (three females and seven males, bodyweight: 25 ± 3 kg, age: 14 ± 2 months at control experiment) received omipalisib at 8:00 AM and 5:00 PM. The compound was administered orally *via* powder-filled capsules (size 0). One dog received no omipalisib capsule on day 7 due to severe adverse events. Venous blood samples were taken *via* the cephalic vein on day 1 and day 7 (1 h after administration) for once-daily dosing, and on day 1 (1, 4, 8, and 24 h after administration) and day 7 (1 h after administration) for twice-daily dosing. The welfare and body weight (BW) of the animals were checked daily.

### Induction experiment

The second experiment under general anesthesia included testing of the arrhythmic status of the heart after omipalisib treatment (Figure 1). The procedure was performed on day 7 of omipalisib treatment, corresponding to 15 ± 1 days after the control experiment. Similar to the control experiment, the arrhythmic status of the heart was tested under anesthesia, bradycardia (the pacing rate at VVD mode was reduced to VVI 60), and a dofetilide challenge. At the end of the procedure, heparin (10.000 IU, i.v.) was infused and hearts were excised (of dogs dosed twice/day) by right-sided thoracotomy, weighed, and used for single cell isolation.

### Data acquisition

Experiments included a continuous recording of the surface electrocardiogram (ECG) and endocardial LV and RV monophasic action potential (LV and RV MAP) signals (catheter from Hugo Sachs Elektronik, Germany). LV pressure (LV-P) signals were acquired for three dogs with a 7F pressure catheter (CD Leycom Inc., Zoetermeer, The Netherlands). All signals were acquired at a sampling rate of 1 kHz using EP-Tracer software (Cardiotek, Maastricht, The Netherlands).

### Analysis of electrophysiological and pressure signals

ECG parameters were measured from five consecutive complexes on lead II of the surface ECG. The RR, PP, QRS, and QT intervals were determined via offline analysis by manually setting the markers in EP-Tracer. Ten consecutive complexes were included for RR and PP interval analysis from awake dogs. The JT interval was obtained by subtracting the QRS interval from the QT interval, and the QT corrected for heart rate (QTc) was calculated using the Van de Water formula (30). The LV MAP and RV MAP duration (LV MAPD, RV MAPD) at 80% of repolarization of five consecutive action potentials were measured semi-automatically using custom-made software (AutoMAPD, MATLAB, Mathworks, Natick, MA, USA). Temporal dispersion or beat-to-beat variation of repolarization was quantified as short-term variability (STV) of repolarization and was calculated from 30 consecutive beats using the formula:


$$\text{STV}=\sum \left|{\text{D}}_{\text{n+1}}-{\text{D}}_{\text{n}}\right|/\left[30^{*}\sqrt{2}\right]$$

with D referring to LV MAPD and RV MAPD (31). Ectopic beats and complexes with a P-wave in the T-wave end were excluded.

The maximum of the derivative of the LV pressure signal, referring to the point where the slope of the pressure rise is the steepest (LVdP/dt_max_), was manually measured offline using AutoMAPD. The mean LVdP/dt_max_ was based on five consecutive pressure cycles. The duration of five consecutive LV-P cycles was manually measured in EP tracer by selecting the start of the QRS on the surface ECG and the end of the pressure cycle (QLVP_end_). Calculation of the electromechanical window (EMW) was performed by subtraction of the QT interval from the QLVP_end_ (32). The maximum and minimum of the derivative of the LV-P signal, referring to the point where the slope of the pressure rise and fall is the steepest (LVdP/dt_max_ and LVdP/dt_min_, respectively), were manually measured offline using AutoMAPD (MATLAB, Mathworks, Natick, MA, USA). The mean LVdP/dt was based on five consecutive LV-P cycles. Time point baseline corresponds to the complexes before the onset of dofetilide infusion. Time point dofetilide corresponds to 5 min after the onset of infusion, or the occurrence of an ectopic beat within these 5 min (for ECG parameters: *n* = 1, and for MAPD and STV parameters: *n* = 6). Signals that were invalid for reliable analysis were excluded.

### Quantification of arrhythmic outcome

The arrhythmia score (AS) is a quantification of the severity of the arrhythmic outcome (33, 34), and is the average of the three highest scored arrhythmic events that occurred in 10 min after the onset of dofetilide infusion. A regular beat is scored with 1 point, single ectopic beats (sEB) are scored with 2 points, multiple ectopic beats (mEB) are scored with 3–5 points, and TdP arrhythmias from 6 complexes or more are scored with 6–49 points. TdP arrhythmias lasting longer than 10 s were defibrillated and scored with 50, 75, and 100 points depending on the number of shocks that were required.

###  Quantification of omipalisib in blood plasma

Blood samples were centrifuged at 4,696 g for 5 min at 4°C and plasma was stored at −80°C until further analysis. Omipalisib concentration in plasma was determined using protein precipitation and liquid chromatography-tandem mass spectrometry (LC-MS/MS) as previously described (35).

### Cellular electrophysiology

Hearts from omipalisib-treated dogs (*n* = 7, dosed twice-daily, three females and four males, bodyweight: 21 ± 2 kg, age: 15 ± 3 months at day of heart isolation) and healthy surplus beagle dogs (*n* = 6, three females and three males, bodyweight: 10 ± 2 kg, age: 1–3 years, Charles River Laboratories, ‘s-Hertogenbosch, The Netherlands) were excised and put on a Langendorff system *via* ligation of the left circumflex coronary artery and right coronary artery. A transmural biopsy of the LV apex was obtained from the omipalisib-treated dogs and immediately frozen using liquid nitrogen for protein analysis. LV and RV cardiomyocytes were enzymatically isolated and stored as previously described (36). Cells were stored at room temperature (RT) in 0.2 mM Ca^2+^ standard buffer solution (in mM: 130 NaCl, 5.4 KCl, 1.2 KH_2_PO_4_, 1.2 MgSO_4_, 6 HEPES and 20 glucose, pH 7.2 corrected with NaOH) until used for whole-cell patch clamp experiments at the same day. Action potentials and potassium currents were recorded using Clampex 10 software (Molecular Devices, Sunnyvale, CA, USA). Action potentials were analyzed using Peaks, a custom Matlab script, which is freely available through the Open Science Framework (https://osf.io/86ufe/). Potassium currents were analyzed using Clampfit 10 software (Molecular Devices, Sunnyvale, CA, USA).

For action potential recordings, cells from SR (*n* = 3) and omipalisib-treated dogs (*n* = 3) were stimulated with 2 ms current injections at 0.5 Hz in a temperature-controlled chamber perfused with (in mM) 137 NaCl, 5.4 KCl, 0.5 MgCl_2_, 1.8 CaCl_2_, 11.8 HEPES and 10 glucose (pH 7.4) at 37°C. Patch pipettes with a 1.5-2.5 MΩ resistance were filled with (in mM) 10 NaCl, 130 KCl, 0.5 MgCl_2_, 5 MgATP and 10 HEPES (pH 7.2). Action potentials after 10 min of stable recording were used to obtain action potential duration (APD) at 90% of repolarization and STV.

For potassium current measurements, cells from SR (*n* = 3) and omipalisib-treated dogs (*n* = 4) were perfused with bath solution containing (in mM) 145 NaCl, 4 KCl, 1 MgCl_2_, 1.8 CaCl_2_, 10 HEPES, 11 glucose and 0.005 nifedipine (pH 7.4) at 37°C. Pipette solution consisted of (in mM) 20 KCl, 1 MgCl_2_, 5 MgATP, 5 HEPES, 125 K-aspartate and 10 EGTA (pH 7.2). Cells were kept at a holding potential of −80 mV and a voltage protocol was applied that consisted of the following steps: −80 mV for 100 ms, −40 mV for 500 ms, −20 to +60 mV with 10 mV increments for 2,000 ms, −50 mV for 1,000 ms, −80 mV for 150 ms, −100 mV for 200 ms and −80 mV for 100 ms. After recording the total current (I_K_), the I_Ks_-sensitive current was determined by adding 500 nM HMR 1556 followed by the addition of 1 μM dofetilide to obtain the I_Kr_-sensitive current. The interval between the addition of the two inhibitors was ±3 min. Current levels were determined as peak tail-current at −50 mV (after the 60 mV step pulse) and corrected for the cell capacitance to obtain current densities.

### Immunoblotting

Frozen cardiac biopsies were pulverized using a liquid nitrogen-cooled mortar and lysates were made using RIPA buffer: (in mM) 20 Tris, 150 NaCl, 10 Na_2_HPO_4_.2H_2_O, 1% Triton X-100, 1% Na-deoxycholate, 0.1% SDS, 1 Na_2_EDTA, 50 NaF (pH 7.4) supplemented with 1 mM phenylmethylsulfonyl fluoride (PMSF) and 10 μg/ml aprotinin. Protein lysates prepared in Laemmli sample buffer were incubated for 5 min at 37°C and 30 μg was separated by 10% SDS-PAGE and blotted on a nitrocellulose membrane. Equal protein loading was revealed by ponceau S staining. Membranes were blocked with 5% BSA in Tris-buffered saline/ Tween 20 (20 mM Tris-HCl pH 8.0, 150 mM NaCl, 0.05% Tween-20) for 1.5 h at RT. Phospho-Akt protein was detected by incubation with polyclonal anti-pAkt primary antibody (1:1000, Cell Signaling Technology, Danvers, Massachusetts, USA) overnight and HRP-conjugated anti-rabbit secondary antibody (1:7000, Bio-Rad Laboratories, Hercules, USA) for 2 h at RT. Final detection was performed by a standard chemiluminescence procedure (Cytiva, Amersham, United Kingdom) with ChemiDocXRS system (Bio-Rad Laboratories, Veenendaal, The Netherlands). Signal analysis was performed using Image Lab software (version 6.1, Bio-Rad Laboratories, Veenendaal, The Netherlands).

### Statistical analysis

Data are presented as mean ± standard deviation (SD). Data were analyzed using a paired Student's *t*-test, (repeated measures) one-way analysis of variance (ANOVA) with Tukey's multiple comparisons test, or a repeated measures two-way ANOVA with Tukey's or Bonferroni's multiple comparisons test. Statistical analyses were performed with GraphPad Prism (version 9.3.1, GraphPad Software, San Diego, USA). A value of *p* < 0.05 was considered statistically significant.

## Results

### ECG parameters and dose testing after once daily omipalisib

**Figure 2:**
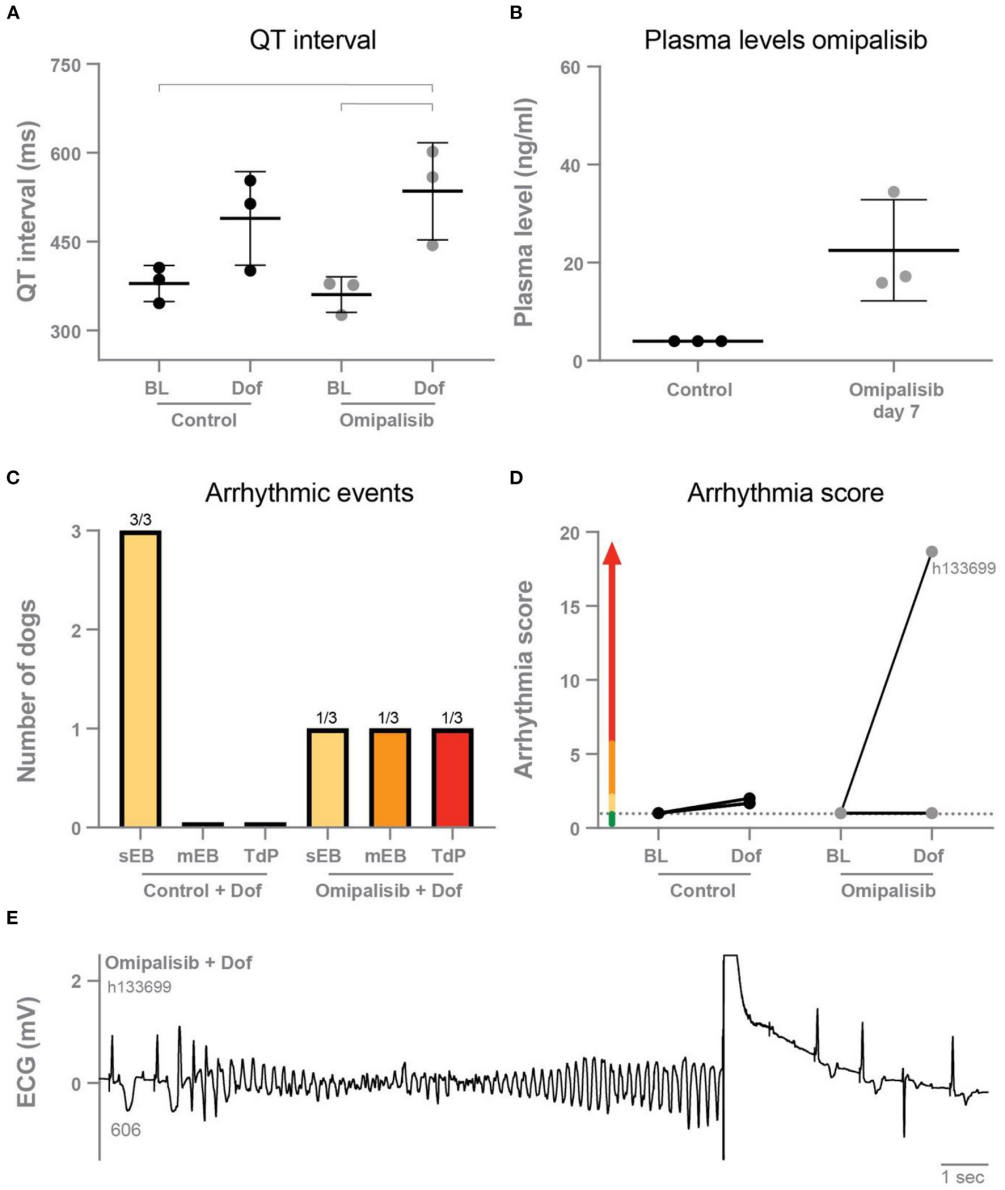
**(A)** QT interval during baseline and dofetilide before treatment (control) and after omipalisib treatment (1 mg/kg once a day). Repeated measures two-way ANOVA with Tukey's multiple comparisons, bars refer to *p* < 0.05. **(B)** Plasma levels of omipalisib before (values <4 ng/ml) and after 7 days of omipalisib treatment. Data are presented as mean ± SD. **(C)** Incidence of arrhythmic events. **(D)** Arrhythmia score of dogs before (control) and after 1 mg/kg omipalisib once a day. Single ectopic beats (sEB, scored with 2 points), multiple ectopic beats (mEB, scored with 3–5 points), and Torsade de Pointes (TdP) arrhythmia (scored with 6–49 points). TdP arrhythmia demanding defibrillation was scored with 50, 75 or 100 points depending on the number of shocks. **(E)** ECG lead II showing a defibrillated TdP arrhythmia after omipalisib treatment and dofetilide infusion in dog h133699. The QT interval (ms) of the complex before the TdP is stated below the T wave.

Omipalisib was administered once a day to three dogs for 7 days to determine plasma levels and its effect on electrophysiological parameters and arrhythmic outcome. The ECG parameters under anesthesia during baseline and after dofetilide, before (control) and after omipalisib treatment are presented in Table 1. Though not significant, dofetilide prolonged the QT and JT interval during the control experiment (Table 1; Figure 2A). These repolarization intervals were significantly prolonged by dofetilide after chronic omipalisib treatment. During the control experiment, solely ectopic beats occurred in all animals during the dofetilide challenge, whereas ectopic beats perpetuated in multiple ectopic beats and TdP arrhythmia in one animal after omipalisib treatment (Figure 2C). The AS increased from 1 to 18.7 for this dog (Figure 2D) due to the sudden-onset TdP arrhythmia demanding defibrillation (Figure 2E), while the AS was unaltered for the other two animals (Figure 2D). Accumulative omipalisib levels in plasma on day 7 of treatment increased to 15.9−34.5 ng/ml and were below the target concentration of 30 ng/ml for two out of three dogs (Figure 2B).

**Table 1:** Electrophysiological parameters of anesthetized dogs (*n* = 3) before treatment (control) and after 1 mg/kg omipalisib treatment for once a day.

	**Control**		**Omipalisib**	
**Parameter**	**Baseline**	**Dofetilide**	**Baseline**	**Dofetilide**
RR	1,000 ± 0	1,000 ± 0	1,000 ± 0	1,000 ± 0
PP	574 ± 46	700 ± 46	416 ± 67	535 ± 67
QRS	113 ± 3	114 ± 2	112 ± 4	114 ± 5
QT	379 ± 30	489 ± 79	361 ± 30	535 ± 82^*#^
JT	266 ± 31	375 ± 77	248 ± 29	423 ± 80^*#^

Parameters in ms. Data as mean ± SD. Repeated measures two-way ANOVA with Tukey's multiple comparisons test.

^*^p < 0.05 compared to control baseline and ^#^p < 0.05 compared to omipalisib baseline.

### Adverse events and increased plasma levels after twice daily omipalisib

**Figure 3:**
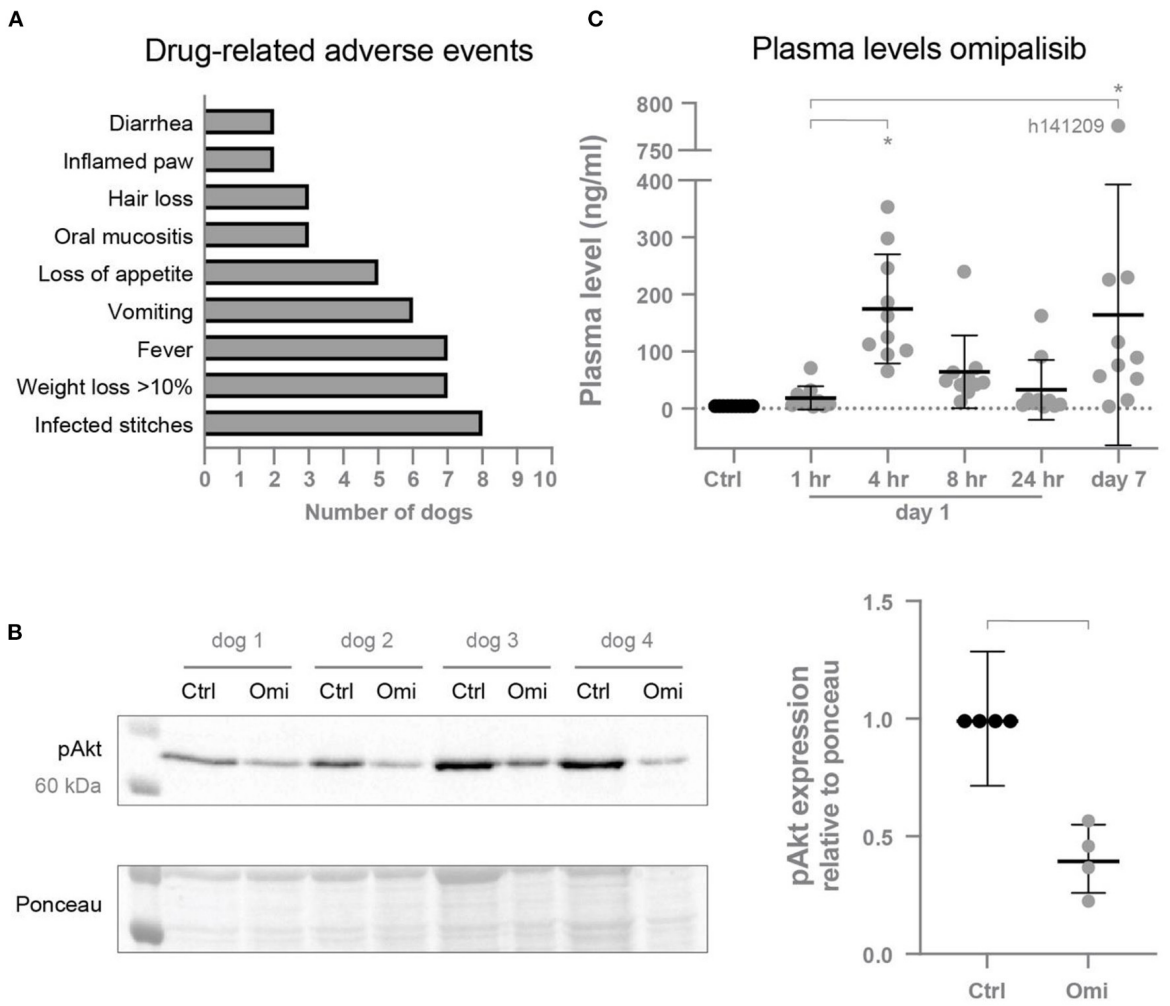
**(A)** Drug-related adverse events induced by omipalisib. **(B)** Reduced pAkt protein expression relative to ponceau after omipalisib treatment (Omi) compared to control (Ctrl) measured in cardiac biopsies. Paired *t*-test, bar refers to *p* < 0.05. **(C)** Levels of omipalisib in blood plasma from dogs (*n* = 10) treated with 1 mg/kg omipalisib twice a day. Repeated measures one-way ANOVA with Tukey's multiple comparisons test, *p < 0.05 compared to Ctrl and for bars. Data are presented as mean ± SD.

Omipalisib was administered twice a day to ten dogs to examine if this higher dose would increase plasma levels to its target levels and the risk for proarrhythmic conditions. Drug-related adverse events were determined, with infected stitches, fever and weight loss as the most common reactions (Figure 3A). Inhibition of the PI3K/mTOR pathway by omipalisib was confirmed by reduced pAkt protein levels in isolated ventricular biopsies (Figure 3B). The weight of the isolated hearts (HW) of omipalisib treated dogs was 238 ± 32 g and the HW/BW corrected for omipalisib-induced weight loss (by taking the BW before treatment) was 9.6 ± 1.3 g/kg. Indeed, on day 1 at 4 h after administration of omipalisib, the accumulative omipalisib levels in plasma were >30 ng/ml in all dogs and maintained above this target concentration at 8 h after the first capsule in 80% of the dogs (Figure 3C). Accumulative omipalisib levels at the last day of treatment were 164 ± 229 ng/ml, with levels above the target concentration in 80% of the dogs (Figure 3C).

### Repolarization duration is not affected by VDD pacing and omipalisib treatment in awake dogs

Alternative to the physiological path of ventricular conduction through the septum in SR, pacing at VDD mode (to maintain ventricular rate at SR) initiated ventricular conduction from the right ventricular apex. Accordingly, the possible effect of RVA-VDD pacing and omipalisib treatment was examined by analyzing ECG parameters of awake dogs through the experimental protocol (Table 2). Both interventions did not affect the RR interval but prolonged the QRS, QT, and QTc intervals compared to SR. The JTc interval, reflecting the repolarization duration by exclusion of the QRS duration from the QTc interval, was not affected by pacing and omipalisib treatment.

**Table 2:** Effect of RVA-VDD pacing and omipalisib treatment on electrophysiological parameters in awake dogs (*n* = 9).

	**No treatment**			**Omipalisib**
	**Sinus rhythm**	**RVA-VDD pacing**		
**Parameter**	**Day−2**	**Day 1**	**Day 8**	**Day 14**
RR	604 ± 94	653 ± 102	535 ± 94	582 ± 138
QRS	77 ± 3	124 ± 9^*^	116 ± 8^*^^∧^	121 ± 9^*^
QT	212 ± 8	277 ± 10^*^	248 ± 13^*^^∧^	267 ± 9^*#^
QTc	247 ± 5	308 ± 13^*^	290 ± 6^*^^∧^	302 ± 12^*^
JTc	170 ± 4	184 ± 16	174 ± 7	181 ± 15

Parameters in ms. Data as mean ± SD. Repeated measures one-way ANOVA with Tukey's multiple comparisons test, ^*^p < 0.05 compared to sinus rhythm at day−2, ^∧^p < 0.05 compared to RVA-VDD pacing day 1, and ^#^p < 0.05 compared to RVA-VDD pacing day 8. Parameters of one dog were excluded because of pacing defects at day 14.

### Twice daily omipalisib prolongs repolarization duration

**Figure 4:**
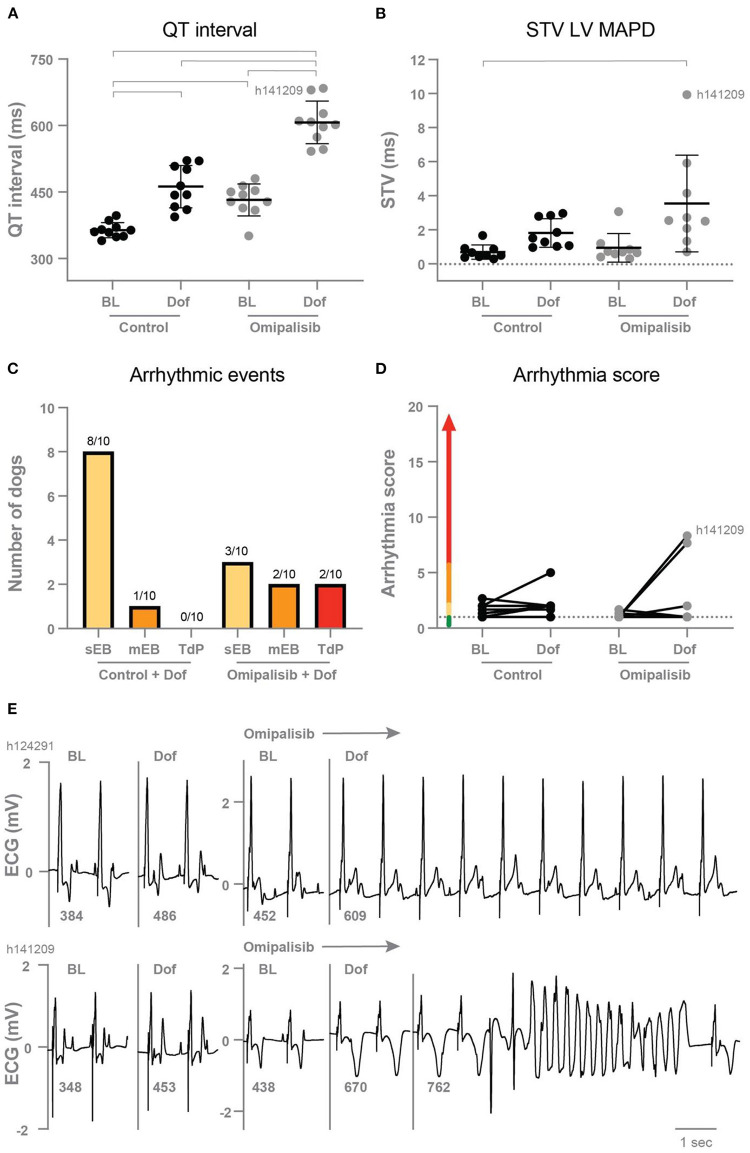
**(A)** QT interval (*n* = 10) and **(B)** short term variability of repolarization (STV LV MAPD, *n* = 9) in dogs before (Control) and after 1 mg/kg omipalisib twice a day during baseline (BL) and dofetilide (Dof). Data are presented as mean ± SD. Repeated measures two-way ANOVA with Tukey's multiple comparisons test, bars refer to *p* < 0.05. **(C)** Incidence of arrhythmic events and **(D)** arrhythmia score during 10 min after start of Dof before and after omipalisib treatment. Single ectopic beats (sEB, score: 2 points), multiple ectopic beats (mEB, scored with 3–5 points), and Torsade de Pointes (TdP) arrhythmia (scored with 6–49 points). TdP arrhythmia demanding defibrillation were scored with 50, 75 or 100 points depending on the number of shocks. **(E)** ECG lead II of the two dogs: h124291 with arrhythmia score of 1, and h141209 with a self-terminating TdP arrhythmia and arrhythmia score of 8.3.

In the control experiment, dofetilide prolonged the PP interval and all (intra)cardiac repolarization parameters (QT, JT, LV MAPD, and RV MAPD, Table 3). Interestingly, chronic omipalisib treatment prolonged repolarization parameters QT, JT and RV MAPD during baseline and more strongly during dofetilide for all repolarization parameters (Table 3; Figure 4A). Furthermore, the temporal dispersion of repolarization—quantified by the STV—was increased after omipalisib treatment combined with dofetilide (Table 3; Figure 4B). A measure of spatial dispersion of repolarization, quantified by the ΔMAPD, remained unaltered upon dofetilide infusion or omipalisib treatment (Table 3).

**Table 3:** Electrophysiological parameters of anesthetized dogs (*n* = 10) before treatment (control) and after 1 mg/kg omipalisib treatment twice a day.

**Parameter**	**Control**		**Omipalisib**	
	**Baseline**	**Dofetilide**	**Baseline**	**Dofetilide**
RR	1,000 ± 0	1,000 ± 0	1,000 ± 0	1,000 ± 0
PP	471 ± 54	615 ± 60^*^	506 ± 64	661 ± 118^*#^
QRS	123 ± 7	125 ± 7	134 ± 9^*^	134 ± 8^*^^∧^
QT	364 ± 17	490 ± 37^*^	432 ± 36^*^	607 ± 48^*^^∧#^
JT	241 ± 15	365 ± 39^*^	298 ± 29^*^	473 ± 48^*^^∧#^
LV MAPD	248 ± 21	352 ± 52^*^	282 ± 18	459 ± 54^*^^∧#^
RV MAPD	222 ± 25	274 ± 29^*^	271 ± 13^*^	408 ± 35^*^^∧#^
ΔMAPD	25 ± 15	75 ± 49	17 ± 12	41 ± 25
STV LV MAPD	0.70 ± 0.42	1.83 ± 0.84	0.94 ± 0.84	3.54 ± 2.84^*^
STV RV MAPD	0.62 ± 0.24	1.13 ± 0.64	0.83 ± 0.49	5.26 ± 4.42^*^^∧#^

Parameters in ms. Data as mean ± SD. PP interval: n = 9. Monophasic action potential duration from the left ventricle (LV MAPD, n = 9) and right ventricle (RV MAPD, n = 8) at 80% of repolarization. ΔMAPD = LV MAPD – RV MAPD. STV = short term variability. Repeated measures two-way ANOVA with Tukey's multiple comparisons test, ^*^p < 0.05 compared to control baseline, ^∧^p < 0.05 compared to control dofetilide, and ^#^p < 0.05 compared to omipalisib baseline.

### Omipalisib has a mild proarrhythmic outcome in the AV block dog model

After omipalisib treatment and the dofetilide challenge, 30% of the dogs showed sEB which perpetuated in mEB and TdP in 20% of the dogs (Figure 4C). The TdP arrhythmias that occurred in the 10-min time window after the onset of dofetilide included 15 self-terminating TdP arrhythmias scored with 6–9 points for one dog and a self-terminating TdP scored with 17 points for the other dog (h141209) (Figure 4E). Their arrhythmia score increased from 1 to 7.7 and 1 to 8.3 (Figure 4D) and both dogs showed the highest omipalisib plasma levels in plasma on day 7. The proarrhythmic effect of an additional trigger in the form of enhanced contractility was examined supplementary to omipalisib treatment. Contractility was enhanced by Na^+^/K^+^-ATPase pump inhibitor ouabain in three dogs from the twice-daily omipalisib-treated group (Supplementary Table 1; Supplementary Figure 1B). However, the ouabain challenge did not increase the proarrhythmic outcome (Supplementary Figures 1C,D).

### Diminished I_Ks_ density in isolated cardiomyocytes from omipalisib-treated dogs

**Figure 5:**
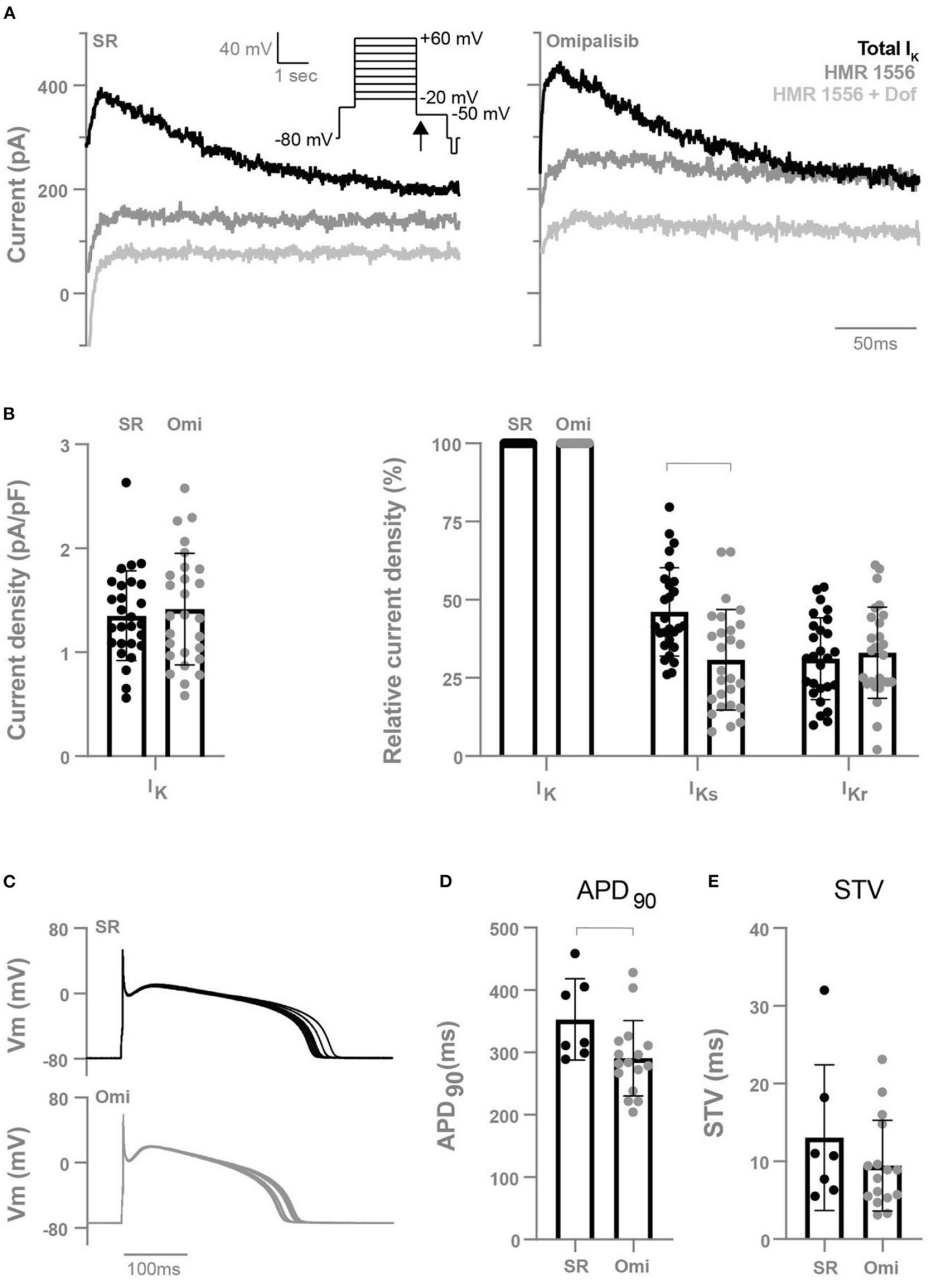
Electrophysiological measurements of isolated cardiomyocytes from the left and right ventricle of dogs in sinus rhythm (SR) and after 1 mg/kg omipalisib twice a day (Omi). **(A)** Representative peak tail current tracings at −50 mV (arrow). **(B)** Current density of total I_K_ (left graph, *n* = 27, unpaired *t*-test, not significant) and relative current density of I_Ks_ and I_Kr_ to total I_K_ (right graph, *n* = 27, repeated measures two-way ANOVA with Bonferroni's multiple comparisons test SR vs. Omi, bar refers to *p* < 0.05). I_Ks_ refers to the current blocked by 500 nM HMR 1556 and I_Kr_ refers to the current blocked by 1 μM dofetilide (Dof). **(C)** Representative action potential tracings. Membrane potential (Vm). **(D)** Action potential duration at 90% of repolarization (APD_90_) and **(E)** short term variability (STV) of repolarization at SR (*n* = 7) and Omi (*n* = 16). Unpaired *t*-test, bar refers to *p* < 0.05. Data are presented as mean ± SD.

To determine the effect of chronic omipalisib treatment on the different potassium currents, electrophysiological measurements were performed on isolated cardiomyocytes from the LV and RV. Representative potassium current tracings from cells of healthy and omipalisib-treated dogs are presented in Figure 5A. Relative to the total I_K_, cells from omipalisib-treated dogs showed a reduced I_Ks_ (Figure 5B). Representative APD tracings are depicted in Figure 5C, and APD at 90% of repolarization of cells from omipalisib-treated dogs (291 ± 61 ms) was marginally shorter compared to SR cells (353 ± 65 ms, *p* < 0.05) (Figure 5D). The APD at 50% of repolarization was 253 ± 58 ms in cells from omipalisib-treated dogs and 317 ± 61 ms in SR cells (*p* < 0.05). The STV of the isolated single cells was not altered after omipalisib (Figure 5E).

## Discussion

This study presents the electrophysiological effects of chronic treatment with the PI3K/mTOR inhibitor omipalisib in a preclinical model. Twice daily dosing was required to reach the omipalisib target concentration. Right ventricular pacing at SR rate and omipalisib did not affect repolarization duration in an awake situation. Yet, omipalisib induced repolarization prolongation under standardized conditions in baseline which further increased after a dofetilide challenge. Furthermore, cardiomyocytes isolated from omipalisib-treated dogs showed a diminished I_Ks_ density. Electrical remodeling induced by omipalisib triggered the occurrence of ectopic beats in 30% of dogs which perpetuated in multiple ectopic beats and TdP arrhythmias in 20% of dogs, resulting in a mild proarrhythmic outcome.

### Role of cardiac remodeling in this study

Cardiac remodeling of the chronic AVB dog model is characterized by bradycardia-induced adaptation on a structural, electrical, and contractile level (37). In the current study design, we prevented these elements of remodeling from occurring by pacing the RV apex at VDD mode to maintain SR rate. A limitation of this pacing protocol is a change in the physiological ventricular conduction pathway causing asynchronous contraction of the ventricles. This can result in LV systolic dysfunction and an increased risk for heart failure (38). Continuation of SR conduction following AV block by so-called His bundle pacing is preferred but coincides with implantation difficulties (39). In the current study, pacing the RV apex at VDD mode and eliminating its effects on repolarization duration by ECG analysis served as the optimal approach toward the maintenance of ventricular rate at SR in the AV block dog model. When focusing on the individual remodeling components, omipalisib did not induce structural remodeling in the form of hypertrophy as the HW and HW/BW were lower compared to previously reported results from chronic AVB dogs, and were similar to SR dogs (40, 41). In addition, the contractile parameter LVdP/dt_max_ was unaltered after omipalisib treatment compared to the control timepoint including values lower than those of previously reported results of chronic AV block remodeled hearts (42). At initiation of this study, it was hypothesized that PI3K/mTOR inhibition by chronic omipalisib treatment could replace AV block-induced electrical remodeling (QT prolongation due to diminished I_Ks_ and I_Kr_) by omipalisib. Indeed, omipalisib induced prolongation of the QT and JT intervals at baseline and more strongly after dofetilide under proarrhythmic conditions. Furthermore, cardiomyocytes isolated from hearts of omipalisib-treated dogs showed a diminished I_Ks_ current. These results demonstrate that chronic PI3K/mTOR inhibition indeed induces proarrhythmic cardiac electrical remodeling to an extent that, at least partially, reflects chronic AV-block induced remodeling.

### PI3K inhibition and cardiac electrophysiology

Examination of the role of PI3K inhibition on cardiac electrophysiology is currently predominantly addressed in *in vitro* studies. Isolated cardiomyocytes from healthy dogs incubated by PI3K inhibitors for 2 h showed a prolonged APD_90_ and diminished densities of the two main repolarizing currents (I_Ks_ and I_Kr_) (16). Despite the diminished I_Ks_ found in the current study, we could not confirm APD_90_ lengthening in isolated cells from hearts after chronic treatment of PI3K inhibitor omipalisib. The diminished I_Ca,L_ density in dog cells incubated with PI3K inhibitor nilotinib (16) and in cells isolated from p110α deficient mice (43) could serve as an explanation for the shorter APD of isolated cells from omipalisib-treated dogs. Furthermore, APD_50_ shortening with similar absolute levels as APD_90_ shortening supports this view on a role of decreased I_Ca,L_ densities. Clearly, absence of measuring I_Ca,L_ densities in the isolated myocytes must be regarded as a limitation of the current study. Nevertheless, as is known, (1) the gap between single-cell measurements lacking environmental influences and *in vivo* proarrhythmic conditions (anesthesia and bradycardia), and (2) incubation duration differences between our and previous studies may add to the contrasting outcome of this and earlier studies on APD_90_ in isolated cells.

PI3K signaling is also involved in glucose regulation and inhibition of the pathway is linked to development of hyperglycemia (44). A study in mongrel dogs with diabetes showed prolongation of the QT interval and a diminished I_Ks_ density, which could be prevented by insulin (45). Despite the similar findings on I_Ks_ density and hyperglycemia as common side effect of PI3K inhibition, the lack of assessment of glucose markers in the current study cannot reveal a link between omipalisib-induced effects on glucose metabolism and repolarization prolongation.

In clinical trials, detailed insight into electrophysiological effects of omipalisib is lacking while examination of cardiac toxicity is a standard item of investigation in drug testing. ECG parameters were addressed in one article and, besides a clinically insignificant reduced heart rate, no abnormalities were reported as adverse events (25). Omipalisib was combined with the RAS/RAF/MEK pathway inhibitor trametinib in a phase Ib trial and showed poor tolerability due to overlapping adverse events (24). ECG and echocardiogram were included in the study design, but unfortunately no outcome was reported.

### Mild proarrhythmic outcome: Safe or warning?

Under standardized proarrhythmic conditions—including anesthesia, bradycardia, and a dofetilide challenge—chronic omipalisib treatment induces prolongation of the QT interval of >100 ms and increases STV. Current guidelines in evaluating drug safety focus on I_Kr_ block and QT prolongation, while compounds can affect multiple ion channels as is shown for omipalisib. STV was suggested as a parameter superior to QT interval prolongation in evaluating the proarrhythmic risk of compounds as established in the AV block dog model (46, 47). Furthermore, 20% of the dogs showed arrhythmic events in the form of ectopic beats perpetuating in self-terminating TdP arrhythmias after omipalisib treatment. Intervening with defibrillation was not required in the animals treated with omipalisib for twice a day, though the initiation of such ventricular arrhythmias can be fatal when sustained. Both dogs with TdP arrhythmias showed the highest omipalisib plasma levels at the induction experiment (day 7), and plasma levels were highly diverse between animals. It should be acknowledged that the accuracy of pharmacokinetics suffers from interindividual (48) and interspecies variability (49). Omipalisib plasma concentrations were compared to target concentrations found in clinical trials, in which plasma level variability and similar adverse events were determined (23). Obviously, the effectiveness of anti-cancer treatment should be weighed against the accompanied cardiotoxic effects. This, with a special focus on cardioprotection in patients susceptible to the development of cardiac disease based on additional risk factors, comorbidity, and genetic profile (10). Integration of knowledge in the cardiology and oncology field is crucial here.

## Study limitations

As a first approach, three dogs received omipalisib once a day to determine its effect on electrophysiological parameters and if target levels were reached. The small sample size may have affected the reliability of the described findings. Furthermore, inhibition of the PI3K pathway affects many more cellular processes than solely cardiac electrical remodeling. Underlying mechanisms resulting from drug-induced adverse effects and potential omipalisib metabolites may have interfered in the study. In addition, the effect of the drug-drug interaction between omipalisib and dofetilide cannot be excluded.

## Data availability statement

The raw data supporting the conclusions of this article will be made available by the authors, without undue reservation.

## Ethics statement

The animal study was reviewed and approved by Experiments were approved by the Central Authority for Scientific Procedures on Animals (Application Approval Number: AVD115002016531), Utrecht University, The Netherlands.

## Author contributions

JB, CP, and MV developed the study design. JB, CP, HB, MHo, AB, and RS performed experiments and contributed to data analysis. JB and MHe wrote the manuscript. MV and MHe provided guidance during the research process. All authors read the paper and approved the manuscript.

## Conflict of interest

The authors declare that the research was conducted in the absence of any commercial or financial relationships that could be construed as a potential conflict of interest.

## Publisher's note

All claims expressed in this article are solely those of the authors and do not necessarily represent those of their affiliated organizations, or those of the publisher, the editors and the reviewers. Any product that may be evaluated in this article, or claim that may be made by its manufacturer, is not guaranteed or endorsed by the publisher.

## Abbreviations

APD: Action potential duration
AV: Atrioventricular block
EMW: Electromechanical window
HW/BW: Heart weight/body weight
I_Kr_: Rapid component of the delayed rectifier potassium outward current
I_Ks_: Slow component of the delayed rectifier potassium outward current
LV-P: Left ventricular pressure
MAP: Monophasic action potential
mEB: Multiple ectopic beat
QLVP_end_: Interval between Q on ECG and end of LV-P cycle
RV: Right ventricular
sEB: Single ectopic beat
STV: Short-term variability
TdP: Torsade de Pointes.
