# Supplementary material for: PI3K/mTOR inhibitor omipalisib prolongs cardiac repolarization along with a mild proarrhythmic outcome in the AV block dog model
Source: Front Cardiovasc Med. 2022 Aug 3;9:956538. doi: 10.3389/fcvm.2022.956538 (PMC9381882; doi:10.3389/fcvm.2022.956538)
Supplement: Supplementary file 1 [file Data_Sheet_1.pdf]

Supplementary Material

1 Supplementary Figures and Tables

Suppl. table 1. Electrophysiological and contractile parameters of dogs (n=3) before and after 1 mg/kg omipalisib twice a day during baseline followed by dofetilide and ouabain.

	**Control**			**Omipalisib**		
Parameter	**Baseline**	**Dofetilide**	**Ouabain**	**Baseline**	**Dofetilide**	**Ouabain**
RR	1000 ± 0	1000 ± 0	1000 ± 0	1000 ± 0	1000 ± 0	1000 ± 0
PP	496 ± 56	617 ± 89	681 ± 85 *	530 ± 101	613 ± 173	651 ± 170 *
QRS	128 ± 9	128 ± 9	130 ± 11	139 ± 9 *	136 ± 7	136 ± 9
QT	375 ± 19	500 ± 49 *	534 ± 36 *	451 ± 27 *	636 ± 44 *^#	582 ± 10 *#&
JT	248 ± 20	372 ± 55 *	404 ± 46 *	312 ± 19 *	500 ± 49 *^#	446 ± 13 *#
$QLVP_{end}$	470 ± 41	478 ± 48	467 ± 20	424 ± 60	422 ± 51	404 ± 38
EMW	94 ± 52	-22 ± 96 *	-67 ± 54 *^	-27 ± 59 *	-214 ± 16 *^#	-177 ± 31 *$#&
$LVdP/dt_{max}$	1185 ± 500	1443 ± 474	2424 ± 667 *^	1058 ± 145	1489 ± 227 #	2290 ± 244 *#&
$LVdP/dt_{min}$	-1211 ± 430	-1363 ± 282	-1955 ± 422 *	-1028 ± 301	-1391 ± 258	-1939 ± 93 *#

Parameters in ms, except for $LVdP/dt_{max}$ and $LVdP/dt_{min}$ in mmHg/s. Data as mean ± SD. Timepoint ouabain: 15 min after onset of infusion. Repeated measures two-way ANOVA with Tukey's multiple comparisons test. *p<0.05 compared to control baseline, ^p<0.05 compared to control dofetilide, $p<0.05 compared to control ouabain, #p<0.05 compared to omipalisib baseline, and &p<0.05 compared to omipalisib dofetilide.

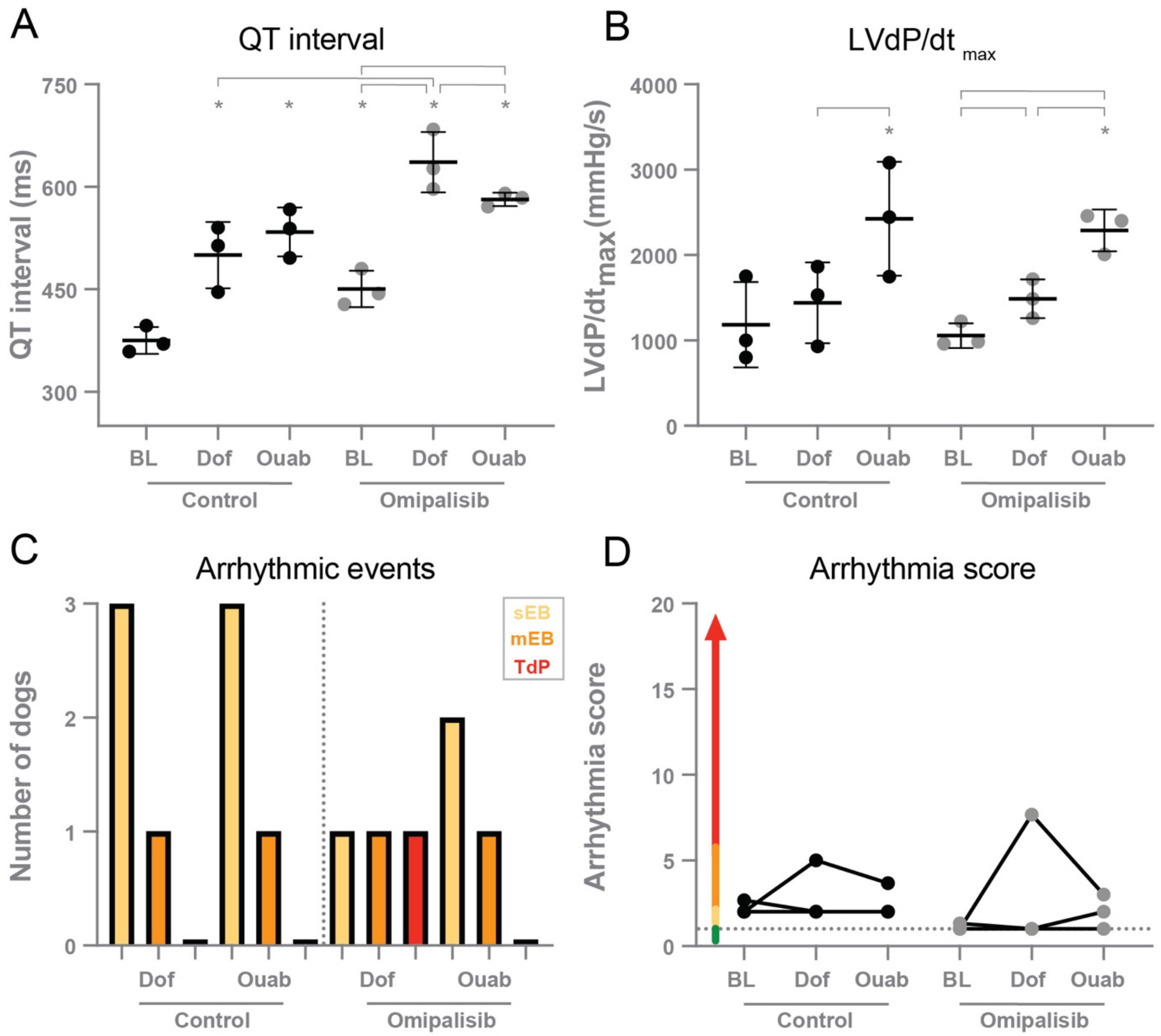

Suppl. figure 1. A) QT interval and **B)** LVdP/dt$_{max}$ of dogs before and after 1mg/kg omipalisib twice a day at baseline (BL), dofetilide (Dof), and 15 min after start ouabain (Ouab). Data are presented as mean ± SD. Repeated measures two-way ANOVA with Tukey's multiple comparisons test. *$p<0.05$ compared to control BL, and for bars. **C)** Incidence of arrhythmic events and **D)** arrhythmia score before and after omipalisib during 10 min after start Dof and 15 min after start Ouab. Single ectopic beats (sEB, score: 2 points), multiple ectopic beats (mEB, scored with 3-5 points), and Torsade de Pointes (TdP) arrhythmia (scored with 6-49 points). TdP arrhythmia demanding defibrillation were scored with 50, 75, or 100 points depending on the number of shocks.
